# Chloroquine sensitivity: diminished prevalence of chloroquine-resistant gene marker *pfcrt*-76 13 years after cessation of chloroquine use in Msambweni, Kenya

**DOI:** 10.1186/s12936-015-0850-9

**Published:** 2015-08-22

**Authors:** William Chege Kiarie, Laura Wangai, Eric Agola, Francis T. Kimani, Charity Hungu

**Affiliations:** Institute of Tropical Medicine and Infectious Diseases (ITROMID), PO Box 54840-00200, Nairobi, Kenya; School of Health Sciences, Kirinyaga University College (Constituent College of JKUAT), P.O. Box 143-10300, Kerugoya, Kenya; Kenya Medical Research Institute, Centre for Biotechnology Research and Development (KEMRI, CBRD), PO Box 54840-00200, Nairobi, Kenya

**Keywords:** *Plasmodium falciparum*, Chloroquine resistance, Mutations, Codon 76, Kenya

## Abstract

**Background:**

*Plasmodium falciparum* resistance to chloroquine (CQ) denied healthcare providers access to a cheap and effective anti-malarial drug. Resistance has been proven to be due to point mutations on the parasite’s *pfcrt* gene, particularly on codon 76, resulting in an amino acid change from lysine to threonine. This study sought to determine the prevalence of the *pfcrt* K76T mutation 13 years after CQ cessation in Msambweni, Kenya.

**Methods:**

Finger-prick whole blood was collected on 3MM Whatman^®^ filter paper from 99 falciparum malaria patients. Parasite DNA was extracted via the Chelex method from individual blood spots and used as template in nested PCR amplification of *pfcrt*. *Apo*1 restriction enzyme was used to digest the amplified DNA to identify the samples as wild type or sensitive at codon 76. Prevalence figures of the mutant *pfcrt* 76T gene were calculated by dividing the number of samples bearing the mutant gene with the total number of samples multiplied by 100 %. Chi square tests were used to test the significance of the findings against previous prevalence figures.

**Results:**

Out of 99 clinical samples collected in 2013, prevalence of the mutant *pfcrt* 76T gene stood at 41 %.

**Conclusion:**

The results indicate a significant [χ^2^ test, P ≤ 0.05 (2006 vs 2013)] reversal to sensitivity by the *P. falciparum* population in the study site compared to the situation reported in 2006 at the same study site. This could primarily be driven by diminished use of CQ in the study area in line with the official policy. Studies to establish prevalence of the *pfcrt* 76T gene could be expanded countrywide to establish the CQ sensitivity status and predict a date when CQ may be re-introduced as part of malaria chemotherapy.

## Background

Malaria in the 21st Century continues to pose a significant challenge to the health and well-being of populations living in malaria-endemic regions worldwide. As per the latest estimates, 198 million cases of malaria occurred globally in 2013, leading to 584,000 preventable deaths [1]. The burden exerted by this disease is heaviest on the African continent, where 90 % of the deaths due to malaria globally occurred [2, 3]. Of these, 78 % were children under the age of 5 years [1, 4]. In Kenya, malaria accounted for 8.82 million cases out of the 41.8 million of outpatient cases reported across Kenyan healthcare facilities in the year 2013 [5]. Pregnant women and young children make up the most vulnerable group to the disease, with 3 % of all deaths in children ≤5 years of age attributed to malaria [5, 6].

Accurate diagnosis followed by prompt treatment with efficacious anti-malarial drugs remains an important cornerstone in the fight against the disease [7]. Microscopy is recommended as the standard tool for parasitological confirmation of malaria as it is highly adaptable to the poor and marginalized setting in most malaria-endemic countries where the majority of cases occur [1, 7]. Rapid diagnostic test (RDT) strips are also employed in malaria diagnosis and are based on detection of specific parasite antigens in a patient’s blood sample. With proper training, RDTs are easy to use and sensitive in detecting low parasitaemia [8, 9]. Efforts to fight back the severe socio-economic impact of the disease have in recent decades been hampered by emergence of strains of *Plasmodium falciparum*- and *Plasmodium vivax*-resistant to past and present anti-malarial drugs [10, 11]. Developed in the 1930s as an effective replacement for quinine, chloroquine (CQ) was quickly adopted as the drug of choice against malaria in all malaria-endemic regions globally [12]. Its key selling points were its low cost, low toxicity and high efficacy in clinical cases, which in the decades following its development made the greatest impact in sub-Saharan Africa where morbidity and mortality due to malaria was drastically reduced [13]. Extensive use and availability of CQ as a monotherapy for malaria prophylaxis led to the emergence of CQ-resistant (CQR) *P. falciparum* and *P. vivax* strains [12, 14]. First noted in Southeast Asia and South America in the late 1950s, CQR *P. falciparum* spread out from these foci to cover all malaria-endemic regions globally, emerging in Africa in the 1970s [14]. *Plasmodium vivax* resistance to CQ emerged much later, pointing to a different genetic mechanism in acquisition of the CQR phenotype [15]. CQ targets the parasite haematin detoxification pathway in the parasite digestive vacuole (DV) in a two-pronged attack where it adsorbs onto growing hemozoin polymers and also binds to toxic haematin molecules generated as the parasite digests host haemoglobin resulting in accumulation of toxic haematin in the DV [16, 17]. Genetic cross studies between CQR and CQ-sensitive (CQS) laboratory strains of *P. falciparum* identified a 13 exon gene, *pfcrt*, on chromosome 7 as the candidate gene for the CQR phenotype [18]. The translated product of *pfcrt* is a multi-domain transmembrane protein localized on the parasite DV. It is thought to play a role in maintenance of osmotic balance by shuttling one or more essential osmolytes across the DV membrane [18–20]. To counter CQ, *P. falciparum* is believed to primarily employ a mutant *pfcrt* product to increase efflux of CQ from the DV [20]. This phenotype is the result of complex genetic changes involving point mutations on ten or more codons of the *pfcrt* gene [18, 21, 22]. Numerous studies have identified the K76T mutation as the central factor in CQR development [18, 21, 23]. This mutation is present in all CQR laboratory lines and field isolates from various malaria-endemic regions and can reliably be used as a CQR marker alone [23] or in concert with other *pfcrt* mutations. Other point mutations on *pfcrt* aside from K76T seem only to modulate CQR as determined by the change on codon 76 [23]. The Pgh-1 (P-glycoprotein homologue 1) encoded by the *P. falciparum pfmdr*-*1* gene bearing specific mutations, particularly the N86Y substitution, has been mentioned as a CQR modulator [24, 25]. Statistical associations between N86Y *pfmdr*-1 and CQR were drawn initially but this gene was later shown to bear no independent effect on CQR and neither does it strengthen K76T based CQR [23]. Its role in CQR may however relate to parasite fitness adaptation in response to physiological changes from *pfcrt* point mutations [12].

Therapeutic efficacy studies are critical in guiding drug policy and are recommended every 2 years where possible [1]. In light of emerging resistance to current first line anti-malarial drugs, namely artemisinin, the importance of reconsidering the efficacy of CQ cannot be understated, given the time interval since its withdrawal. Malawi was the first country in Africa to cease administration of CQ in malaria chemotherapy in 1993 following widespread treatment failure [24]. Kenya followed suit in 1999 as did many sub-Saharan countries on the continent [26]. Sulfadoxine-pyrimethamine (SP) replaced CQ as the first-line anti-malarial drug in these countries but was discontinued in 2004 following rapid development of parasite resistance to these drugs and replaced with artemisinin-based combination therapy (ACT) [7]. Where CQ use was effectively discontinued through concerted public awareness efforts, studies have shown that in absence of drug pressure CQR strains of *P. falciparum* with an associated loss in fitness have steadily been replaced by CQ-sensitive (CQS isolates). As has been the case especially in Malawi, where the withdrawal of CQ was supported by an intensive national sensitization programme, the population frequencies of mutations associated with CQR in *pfcrt* have reduced from 85 % in 1992 to 13 % in 2000 [24]. Patterns in CQR reversal heavily depend on national drug policies and public awareness campaigns as evidenced by persistence of CQR strains of *P. falciparum* in certain regions where CQ withdrawal was inconclusive in contrast with regions that efficiently removed CQ out of national circulation [24]. Although the trend has not been as drastic as in Malawi, other reports of significant rises in prevalence of CQS *P. falciparum* isolates have emerged [27]. This reversal to CQS presents an interesting scenario to drug policy makers in these regions where CQ can be re-introduced as malaria chemotherapy, although current knowledge would advocate for CQ administration in concert with another anti-malarial drug that differs in mechanism of action to slow down development of anti-malarial drug resistance [1, 7, 24]. Sixteen years have passed since Kenya withdrew CQ as the first line anti-malarial drug in 1999. A retrospective study done in Kilifi, Kenya [27] reported a *pfcrt* 76T prevalence of 63 %, down from 94 %, in the 13 years that had passed since CQ withdrawal. The current study thus sought to survey the current sensitivity levels of *P. falciparum* to CQ in Msambweni area, coastal Kenya, 16 years after official withdrawal of CQ as the first line of treatment for uncomplicated malaria.

## Methods

### Study site

The study was carried out in the Kenyan coastal constituency of Msambweni, Kwale County. The area is located about 56 km south of Mombasa city and borders Tanzania to the south. The area sits at less than 300 m above sea level and its climate is hot and humid with annual average rainfall between 900 and 1500 mm. Humidity ranges between 70 and 80 % with an annual mean temperature ranging between 22 and 34 °C. According to the 2010 Kenya Malaria Indicator Survey, Kwale County is a malaria-endemic zone with stable *P. falciparum* transmission. Malaria in this area accounts for 40 % of all outpatient visits and 40 % of all inpatient admissions. *Plasmodium falciparum* is transmitted by two main vectors in this area: *Anopheles gambiae* and *Anopheles funestus*. These vectors exist all through the year but their population peaks with the short rains in the months between April and June and October and November.

### Ethical considerations

The study was be approved by the Kenya Medical Research Institute (KEMRI) Nairobi, Ethical Review Committee (ERC). Informed consent was obtained from parents of eligible children aged between 6 months and 10 years before they were enrolled in the study.

### Study population

Patients visiting the outpatient clinic at Msambweni District hospital were enrolled in the study based on the following criteria: consent, mono-infection of *P. falciparum* with parasitaemia between 1000 and 200,000 parasites/µL of blood, axillary temperature ≥37.5 °C, or with a history of fever, and no history of anti-malarial drug intake during the previous week. Patients were excluded from the study if there was administration of any additional anti-malarial drugs, emergence of any non-malarial febrile illness that would interfere with the classification of the treatment outcome, patient relocation from the study site, and withdrawal from the study.

### Experimental design

#### Sample collection

Pre-treatment (day zero) blood samples were collected from patients by finger prick as dried blood spots on 3MM Whatman^®^ filter papers, packaged individually in zip lock bags with a desiccant and transported to KEMRI headquarters in Nairobi, Kenya.

#### *Pfcrt* amplification

DNA extraction was done as described by Warhurst et al. [28]. The amplification of the *pfcrt* gene was done on a GeneAmp^®^ PCR system 9700 machine. PCR amplification was as follows: in a final reaction volume of 25 µL, the outer PCR consisted of 1× PCR buffer (Roche^®^) 1.5 mM MgCl_2_, 200 µM dNTP mix, 100 nM each of CRTP1 (GCGCGCGCATGGCTCACGTTTAGGTGGAG) and CRTP2 (GGGCCCGGCGGATGTTACAAAACTATAGTTACC) and 0.1 µL of Taq polymerase (Bioline^®^, 5 U/µL). The mixture was topped up to a volume of 25 µL with nuclease-free PCR water. The PCR profile was: 95 °C for 5 min, 92 °C for 30 s, 45 °C for 30 s, 65 °C for 45 s, 45 cycles, final extension at 72 °C for 15 min then held at 4 °C. In a final volume of 25 µL the nested PCR consisted of 1 µL outer PCR product, 1× PCR buffer (Roche^®^) 1.5 mM MgCl_2_, 200 µM dNTP mix, 100 nM each of CRTD1 (TGTGCTCATGTGTTTAAACTT) and CRTD2 (CAAAACTATAGTTACCAATTTTG and 0.1 µL of Taq polymerase (Bioline^®^, 5U/µL). The mixture was topped up to a volume of 25 µL with nuclease-free PCR water. The PCR profile was set at 94 °C for 3 min, 94 °C for 30 s, 48 °C for 30 s, 64 °C for 1 min, 30 cycles, final extension at 64 °C for 5 min then halted at 4 °C.

#### Restriction digests of the *pfcrt* gene

Allele specific restriction analysis was done using restriction endonuclease *Apo*1 (New England Bio Labs). Briefly, in a final volume of 20, 1.5 µL of 10× buffer 3, 0.15 µL of bovine serum antigen, 8 µL of amplified DNA, 0.5 µL of *Apo*1 restriction endonuclease was added to 9.85 µL of nuclease-free PCR water. Incubation was done for between 12 and 14 h at 50 °C with no agitation. Digested samples were analysed on a 2 % agarose gel (Bioline^®^) in TAE buffer for 35 min at 80 V, with no-digest controls run in adjacent wells on the gel.

## Results

**Fig. 1:**
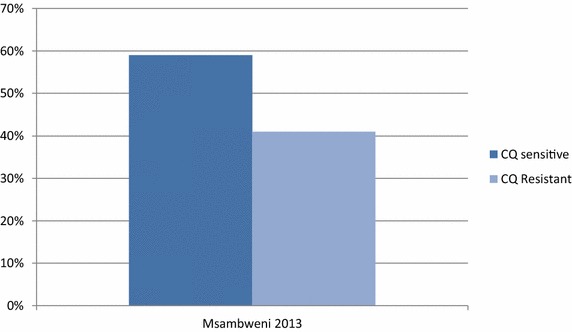
Results obtained after Pfcrt amplicon digestion using *Apo*I restriction enzyme. Of the 99 samples analysed in the study, 59 % (95 % CI: 0.4824–0.6826) were found to be sensitive to *Apo*I digestion, thus sensitive to chloroquine, while 41 % were resistant to chloroquine (95 % CI: 0.3221–0.5126)

A total of 99 samples collected from the Msambweni District Hospital, Kwale County in 2013 were analysed in this study, all of which were from symptomatic, mono-infected microscopy-positive patients. The mean age of the patients, in years, was 4.93 ± SD 2.76 (IQR: 4.64–5.22). Of these, 46.8 % were female and 53.2 % were males. The geometric mean of the asexual microscopic parasite density was 11,813 parasites/µL of blood (95 % CI: 9690–14,402). All the samples run for *pfcrt* gene amplified successfully and had the characteristic 145-bp fragment. Of the positive controls amplified alongside the field samples, the *P. falciparum* W2 clone was resistant to digestion using *Apo*I restriction enzyme while the D6 clone digestion resulted in two bands, approximately 99 and 46 bp long, as expected. These positive controls were used to score the amplified and digested field samples as either mutant or wild type. Using this criterion, 59 % (95 % CI: 0.4824–0.6826) of the samples were sensitive to *Apo*I digestion, thus CQ sensitive, while 41 % (95 % CI: 0.3221–0.5126) of the samples carried the allele conferring resistance to CQ (Fig. 1). In the case of mixed samples harbouring both resistant and sensitive clones, *Apo*I digestion was expected to yield three fragments 145, 99 and 46 bp in length. None of the samples yielded this type of banding pattern thus no mixed samples were identified in this study.

## Discussion

The widespread parasite resistance to CQ on the African continent in the 1990s led to its official ban as a first-line treatment drug for uncomplicated *P. falciparum* malaria in many African countries [24, 26]. The loss of this effective and cheap anti-malarial drug led policy makers to opt for alternative drugs to treat malaria, with SP being the leading candidate for replacement of CQ [24, 27]. Towards the turn of the Millennium, the prevalence of the mutant *pfcrt* allele in Kenya stood at 100 % [29] indicating total failure of CQ as a first-line anti-malarial drug. A key reason behind this treatment failure was the sustained use of CQ as a monotherapy in treatment of uncomplicated malaria for decades since its development [12]. This led to the official ban on CQ as a first-line anti-malarial drug and the subsequent introduction of SP and amodiaquine (AQ), as first- and second-line anti-malarial treatment, respectively, in 1999. Cases of resistance to SP were already emerging in the late 1990s and in the years following its use as the first-line anti-malarial there were widespread reports of *P. falciparum* resistance to SP which necessitated the change in malaria treatment regimen in Kenya in 2004 [30]. Artemether-lumefantrine (AL) was selected as the first-line drug in malaria chemotherapy with oral quinine (QN) being the second-line drug for treatment of uncomplicated falciparum malaria [30, 31]. *pfcrt* mutations conferring CQ resistance have been reported to enhance sensitivity to QN, a feature regarded as a possible exploitable weakness in the CQ resistance mechanism. In 2010 [7] QN was dropped in favour of a safer and less toxic alternative: dihydroartemisinin-piperaquine (DHA-PPQ) as the second-line drug of choice for malaria chemotherapy.

**Fig. 2:**
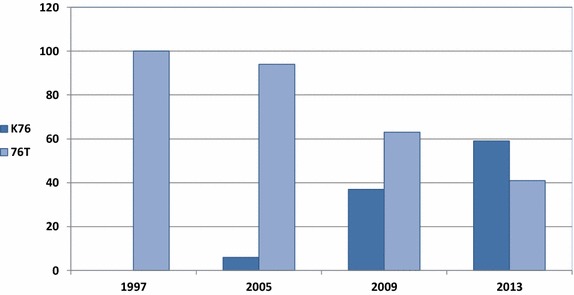
Trends in prevalence of the Pfcrt mutant and wild type alleles across different study sites in Kenya from 1997 through to the current 2013 study. There is a clearly observable reduction in the overall prevalence of the mutant *pfcrt* allele, which is in line with the results obtained in the current study

In this study, the authors sought to establish current status of CQ resistance in Msambweni in the 13 years since CQ was officially banned as malaria chemotherapy. Resistance to CQ is linked to a mutation at codon 76 of the *P. falciparum pfcrt* gene, resulting in an amino acid change from lysine to threonine and this has been proven to be the chief determinant of CQ resistance [18]. Absence of the 76T mutation at this codon has been shown to be a reliable predictor of successful CQ treatment. The current study shows that the prevalence of the mutant *pfcrt* stands at 41 % within a population sampled in 2013, 14 years after official withdrawal of CQ as the first choice in malaria chemotherapy. This decrease is loosely concordant with the Malawian reduction after CQ withdrawal in 1993 for varied reasons [27]. In a 2009 study carried out within Kilifi, the same geographical area as this survey, the prevalence of the mutant *pfcrt* allele was 63 % [27] (Fig. 2). Compared to the current study, this represents a significant (P = 0.0029) drop in the level of *P. falciparum* CQ resistance in the seven-year period between the two studies. Interestingly, the prevalence of the mutant *pfcrt* allele seems to be very discordant (P < 0.0001) across different areas in Kenya in samples analysed between 2005 and 2008. A study conducted in 2005 reported a *pfcrt* 76T prevalence of 94 % in Mwea, central Kenya [32]. Around the same time period, other studies published *pfcrt* 76T prevalence figures of 49 % [33] and 63 % [27] in coastal Kenya and 27.59 % in western Kenya [34]. The stark contrasts noted here could probably be explained by the disparities in enforcement of the ban on CQ as an anti-malarial drug [27]. CQ remained locally available in the years following its official withdrawal as a first-line anti-malarial drug in Kenya [35] although the withdrawal of CQ in the Kilifi area was supported by intensive public awareness programmes as to the diminished efficacy of CQ as an anti-malarial drug [27], and the availability of SP as a more efficacious alternative.

The absence of drug pressure is believed to be a key driver in the resurgence of CQS parasites in the field setting [24]. The mutations that confer *P. falciparum* resistance to CQ come with a significant loss of fitness, causing the CQS parasites to outgrow the mutant strains [24, 32], thus increasing CQ efficacy. Removal of CQ from anti-malarial therapy in Malawi due to widespread treatment failure was followed by a drastic reduction in the prevalence of the mutant *pfcrt* allele from 85 % in 1993 to 13 % in 2000 [24]. Although the reduction in the Kenyan context has not been as dramatic, the 41 % *pfcrt* 76T prevalence reported in this study represents an encouraging scenario where the proportion of CQR strains has been surpassed by the proportion of CQS isolates. The slight but significant drop (P = 0.0289) in prevalence of the mutant *pfcrt* allele from an absolute saturation in 1997 to 94 % (Fig. 2) in a 2005 follow-up study could be attributed to use of AQ as a second-line anti-malarial drug in the official treatment guidelines since AQ selects for *pfcrt* mutant strains. This is in addition to the alleged incomplete withdrawal of CQ from private dispensaries and pharmacies [32].

Although noteworthy, the 41 % prevalence of the mutant *pfcrt* allele still needs further investigation to define the CQ resistance patterns across the country with a view to explain any observed discrepancies in CQ sensitivity levels. The reported 41 % prevalence is based on analysis of only the *pfcrt* gene without comparative prevalence data from the alleged CQR modulator, *pfmdr*-*1* N86Y. The Kilifi report published in 2009 predicted that it would take another 13 years before the CQ resistance levels dipped to a point where policy makers may consider re-introduction of CQ in malaria therapy, albeit in concert with another, different, class of anti-malarial drug. In the 7 years since that prediction was made, there appears to have been a 22 % drop in CQ resistance in the Kilifi area of Kenya. From the results of the current study, it may be predicted that reversion to CQ sensitivity could take longer than initially projected [27] in the Kilifi area. It is possible that sustained presence of the mutant *pfcrt* allele may also be driven in part by genetic compensatory mechanisms in the CQR parasites allowing them to exist in absence of CQ drug pressure.

## Conclusion

The current study has identified an encouraging increase in the prevalence of CQS parasites in the said study area. This could be attributed to a number of factors, chief among which is the reduction or absence of drug pressure to sustain CQR *P. falciparum* strains. Parasite genetic adaptations in absence of drug pressure could also probably explain the sustained presence of the mutant *pfcrt* 76T allele in the population. The World Health Organisation (WHO) set a maximum threshold of 10 % drug resistance before an anti-malarial drug can be banned on the basis of failed efficacy. Although lower than previous years, a *pfcrt* 76T prevalence of 41 % is still much higher than the WHO standard and it would be advisable to investigate if the results of this study can be replicated in other malaria-endemic regions in Kenya. Such investigations could, where resources allow, incorporate other genotyping methods of greater sensitivity [36, 37] in addition to those outlined in this paper to provide a clearer picture of the Kenyan CQR status and test the CQS reversion predictions made previously [27]. This study concludes that there are encouraging indications to suggest the *P. falciparum* resistance to CQ is steadily declining which could be good news for Kenyan drug policy makers as CQ is cheaper and less toxic than other anti-malarial drugs. If CQ sensitivity can be restored in the Kenyan population, this cheap and effective drug can be in future incorporated into anti-malarial therapy in concert with another anti-malarial drug.

## Abbreviations

KEMRI: Kenya Medical Research Institute
CQ: chloroquine
*pfcrt*: *Plasmodium falciparum* chloroquine resistance transporter gene
*pfmdr*-*1*: *Plasmodium falciparum* multidrug resistance-1 gene
SP: sulphadoxine-pyrimethamine
AQ: amodiaquine
AL: artemether-lumefantrine
CQR: chloroquine resistant
CQS: chloroquine sensitive
DHA-PPQ: dihydroartemisinin-piperaquine
dNTP: deoxynucleotide triphosphate
WHO: World Health Organisation
DNA: deoxyribonucleic acid
RDT: rapid diagnostic tests
DV: digestive vacuole
P-GH1: P-glycoprotein homologue 1
